# Internet gambling is a predictive factor of Internet addictive behavior

**DOI:** 10.1556/JBA.2.2013.4.5

**Published:** 2013-12-13

**Authors:** Elena Critselis, Mari Janikian, Noni Paleomilitou, Despoina Oikonomou, Marios Kassinopoulos, George Kormas, Artemis Tsitsika

**Affiliations:** ^1^Second University Department of Pediatrics, “P. & A. Kyriakou” Children’s Hospital, National and Kapodistrian University of Athens School of Medicine, Athens, Greece; ^2^Adolescent Health Unit (AHU), Second University Department of Pediatrics, “P. & A. Kyriakou” Children’s Hospital, National and Kapodistrian University of Athens School of Medicine, Athens, Greece; ^3^Pediatric Society of Cyprus, Nicosia, Cyprus; ^4^Cyprus University of Technology, Nicosia, Cyprus

**Keywords:** adolescent, Internet, gambling, addictive behavior, psychosocial aspects

## Abstract

*Background and aims:* Adolescent Internet gambling is associated with concomitant addictive behaviors. This study aimed to assess the prevalence of Internet gambling practices, its impact upon psychosocial development and to evaluate the association between gambling practices and Internet addictive behavior among Cypriot adolescents. *Methods:* A cross-sectional study was conducted in a convenience sample (*n* = 805) of adolescents attending selected public schools (9th and 10th grades) in Cyprus. Anonymous self-completed questionnaires were used including the Internet Addiction Test and the Strengths and Difficulties Questionnaire. *Results:* Among the study population (*n* = 805), approximately one third (*n* = 28; 34.9%) reported Internet gambling. Internet gamblers were twice as likely to utilize Internet café portals (adjusted odds ratio for gender and age, AOR: 2.13; 95% confidence interval, 95% CI: 1.56–2.91) for interactive game-playing (AOR: 6.84; 95% CI: 4.23–11.07), chat-rooms (AOR: 2.57; 95% CI: 1.31–4.85), and retrieval of sexual information (AOR: 1.99; 95% CI: 1.42–2.81). Among Internet gamblers 26.0% (*n* = 73) reported borderline addictive Internet use and 4.3% (*n* = 12) addictive behavior. Internet gamblers more often had comprehensive psychosocial and emotional maladjustment (AOR: 4.00; 95% CI: 1.97–8.13), including Abnormal Conduct Problems (AOR: 3.26; 95% CI: 2.00–5.32), Emotional Symptoms (AOR: 1.78; 95% CI: 1.02–3.11), and Peer Problems (AOR: 2.44; 95% CI: 1.08–5.48) scores. The multivariate regression analyses indicated that the single independent predictor associated with Internet addictive behavior was Internet gambling (AOR: 5.66; 95% CI: 1.45–22.15). *Discussion:* Internet gambling is associated with addictive Internet use, as well as emotional maladjustment and behavioral problems, among Cypriot adolescents. *Conclusions:* Longitudinal studies are needed to elucidate whether Internet gambling constitutes a risk factor for the development of Internet addictive behavior among adolescents.

## INTRODUCTION

Among youth the prevalence of gambling practices is reported to range between 1.9% (Molde, Pallesen, Bartone, Hystad & Johnsen, 2009) and 15.1% (Moodie & Finnigan, 2006). The adoption of Internet gambling behaviors among adolescents has increased dramatically during recent years following the widespread penetration of Internet use (Griffiths, Parke, Wood & Parke, 2006; Messerlian, Byrne & Derevensky, 2004). The availability of the Internet as a medium for conducting gambling practices among youth is of particular concern (Dickson, Derevensky & Gupta, 2008). Specifically, adolescents engaging in Internet gambling practices are potentially at risk for developing problematic Internet use and Internet addictive behavior (Griffiths, 2003; Griffiths & Barnes, 2008; Griffiths, Wardle, Orford, Sproston & Erens, 2009; Ladd & Petry, 2002).

As a medium the Internet allows for unlimited and readily accessible opportunities to participate in gambling practices. Both the anonymity and proximity of the Internet permit adolescents to circumvent legal prohibitions regarding gambling practices imposed in real-world venues. The frequency of gambling practices, as opposed to monetary sums gambled, has been identified as a predictor of problematic gambling behaviors (Labrie, Kaplan, LaPlante, Nelson & Shaffer, 2008; Nelson et al., 2008). As a result, adolescents, and particularly potentially problematic gamblers (Griffiths, 2003; Griffiths et al., 2006), are at increased risk for developing problematic behaviors due to the compounded effects of the unmitigated accessibility of the Internet and unrestricted frequency of Internet gambling practices (Griffiths, 1999; Holtgraves, 2009; Nelson et al., 2008; Welte, Barnes, Tidwell & Hoffman, 2009).

Adolescents have an augmented likelihood for developing addictive behavioral patterns (Griffiths & Wood, 2000; Pallanti, Bernardi & Quercioli, 2006), including problematic gambling and Internet addictive behavior. Such vulnerability among this particular population group may be partly attributed to adolescents being both prone to adopting risk-taking behaviors and lacking appreciation for the potential adverse effects of such behaviors (Derevensky, Gupta & Winters, 2003). Consequently, adolescents are at particular risk for developing problematic gambling behaviors. The adverse effects of such gambling practices among youth may regard overall psychological maladjustment (Delfabbro, Lahn & Grabosky, 2006), including peer relations (Gerdner & Svensson, 2003) and conduct problems (Barnes, Welte, Hoffman & Dintcheff, 2005; Vitaro, Brendgen, Ladouceur & Tremblay, 2001), particularly among adolescent boys (Martins, Storr, Ialongo & Chilcoat, 2008).

Furthermore, gambling practices via the Internet allow for an early onset of such behaviors, thus augmenting the likelihood and severity of problematic gambling in youth (Dickson, Derevensky & Gupta, 2002; Pallanti et al., 2006) and in consequent adulthood (Johansson & Götestam, 2003). Since the risk of concomitant and/or multiple addictive disorders among adolescents is increased (Pallanti et al., 2006), the likelihood of problematic Internet gambling behavior and Internet addictive behavior (Shapira, Goldsmith, Keck, Khosla & McElroy, 2000) is amplified. To date, the association between utilizing the Internet as a means for satisfying problematic behavioral patterns (Recupero, 2008), such as problematic gambling (Sun et al., 2009), and the consequent development of Internet addictive behavior has not been elucidated (Griffiths, 2003; Mitchell & Wells, 2007; Wölfling, Bühler, Lemenager, Mörsen & Mann, 2009).

The study aim was to assess the prevalence of Internet gambling practices, as well as its impact upon psychosocial development, among adolescents. In addition, the study evaluated the association between the frequency of gambling practices and development of Internet addictive behavior among youth.

## METHODS

### Study design and study population

A cross-sectional study was conducted during the period January 1st 2009 – January 1st 2010. The original study population (*n* = 915) included a convenience sample of students attending Grades 9 and 10 at public junior high and high schools in Nicosia, Cyprus. Exclusion criteria regarding demographic and/or socioeconomic factors were not applied. The response rate among students attending eligible schools was 88.0% and the final study population consisted of 805 adolescents.

### Data collection

Study participants were requested to complete anonymous questionnaires on-site at their respective schools. The questionnaire (Tsitsika et al., 2009a) consisted of the following components: (1) demographic information; (2) Internet gambling practices; (3) characteristics of Internet use; (4) the Internet Addiction Test (IAT) and (5) the Strengths & Difficulties Questionnaire (SDQ).

### Measurements

Assessment of Internet gambling practices was based on self-reported frequency of Internet gambling with (i.e. online poker games) and without (i.e. online games with coupons or Pamble points) monetary awards. For the purposes of the study, the basis of comparison included adolescents who reported either having never gambled or having gambled less than once per week through Internet sites. Adolescents reporting Internet gambling practices at least once per week were characterized as *Internet gamblers*. *Overall Internet gambling* was further specified as either (1) *infrequent Internet gambling:* participation in Internet gambling 1–4 times per week; or (2) *frequent Internet gambling:* engagement in Internet gambling at least 5 times per week.

Length of Internet use was assessed by examining when study participants had first initiated Internet use and was defined as follows: (1) *novel Internet use:* initiation of Internet use during the past year; (2) *recent Internet use:* initiation of Internet use during the past 12–36 months; and (3) *experienced Internet use:* Internet use lasting more than 3 years. The average number of hours of Internet use was assessed separately for weekdays and weekends. The locations of Internet access included Internet access via: (1) one’s own home portal; (2) a friend’s home portal; (3) Internet café portal and (4) cellular phone. The scope of Internet sites accessed included: (1) e-mail correspondence; (2) retrieval of newspapers, journals, and periodicals (mass media); (3) chat room use; (4) interactive role-playing games; (5) retrieval of information pertaining to sexual education and (6) purchases of goods and services.

The IAT was used to evaluate the presence of Internet addictive behavior among study participants (Young, 1998). The IAT evaluates the degree of preoccupation, compulsive use, behavioral problems, emotional changes, and impact upon functionality arising from accessing the internet. The IAT consists of 20 Likert scaled items providing calibrated scores ranging from 1 to 5, where a score of 1 is defined as ‘‘rarely’’ and a score of 5 as ‘‘always’’. The sum of scores may thus range between 20–100, where higher scores (>40) reflect a greater tendency for problematic Internet use. From the total IAT score adolescent Internet use was defined as: (1) *normal Internet use:* IAT scores 20–49; (2) *borderline addictive Internet use:* IAT scores 50–79 and (3) *Internet addictive behavior:* IAT scores 80–100 (Young, 1998).

The SDQ was applied for the assessment of adolescents’ emotional and psychosocial adjustment (Goodman, 1999). The SDQ includes 25 questions with calibrated response scores ranging from 0 to 2. The SDQ is comprised of five calibrated component scores: (1) the *Emotional Symptoms Scale;* (2) *Conduct Problems Scale;* (3) *Hyperactivity Scale;* (4) *Peer Problems Scale* and (5) *Prosocial Scale*. With the exclusion of the Prosocial Scale, the sum (range 0–40) of the remaining SDQ component scores was computed and identified as the *Total Strengths and Difficulties* score. Abnormal *Total Strengths and Difficulties* scores are associated with mental health disorders.

### Statistical analysis

Adolescents who did not participate in Internet gambling were applied as the basis of comparison for all analyses. Categorical and continuous variables were compared between groups with the chi-square test and student’s *t*-test for independent samples, respectively. The likelihood ratio was used to compare ordinal variables. Logistic regression was used to derive age and gender adjusted odds ratios (AOR) and 95% Confidence Intervals (95% CI) for the locations of Internet access, scopes of Internet sites accessed, and SDQ component and total scores. Stepwise multivariate logistic regression was used to identify factors independently associated with the occurrence of Internet addictive behavior. Independent variables included in the modeling procedure were selected demographic variables, characteristics of Internet use (including length of Internet use, locations of Internet access, and scopes of sites accessed), and Internet gambling. The Hosmer and Lemeshow Goodness of Fit test was used to assess the derived regression model. All analyses were conducted with SAS version 9.0 (SAS Institute Inc., USA) software.

### Ethics

The study protocol was approved by the Ethics Committee of the Hellenic Ministry of Education and Religious Affairs. Informed consent for study participation was granted from the legal guardians of eligible participants.

## RESULTS

### Internet gambling practices among adolescents

The prevalence of Internet gambling among the study sample (*n* = 805) was 34.9% (*n* = 281). Moreover, 21.3% (172) reported infrequent Internet gambling, while 13.5% (*n* = 109) of adolescents reported frequent gambling. Adolescents participating in Internet gambling were more likely to be male and of younger age as compared to their normal counterparts (Table 1).

Adolescents who participated in Internet gambling did not differ from their normal counterparts with respect to any of the demographic characteristics assessed. However, members of this group more often engaged in Internet use both during the weekdays and weekends (Table 2).

Internet gambling was associated with accessing the Internet via either an Internet café portal or one’s cellular phone. While Internet gambling was not associated with Internet use for the purposes of email and/or retrieval of news feeds, it was significantly associated with interactive game playing, chat-room use, and utilization of the Internet for the retrieval of sexual information (Table 3).

Approximately one quarter (26.0%; *n* = 73) of adolescents who participated in Internet gambling concomitantly reported borderline addictive Internet use. Moreover, 4.3% (*n* = 12) of this group were identified with Internet addictive behavior (Table 2). Adolescent Internet gambling was associated with an increased risk of abnormal Total Strengths & Difficulties score. In particular, adolescents reporting Internet gambling practices were more likely than their normal counterparts to present with abnormal conduct problems, peer problems, and emotional symptoms (Table 4).

### Infrequent Internet gambling practices among adolescents

Approximately one fifth (21.3%; *n* = 172) of the study sample reported infrequent gambling practices. Adolescents engaging in infrequent gambling practices more often engaged longer in Internet use either during the weekdays or on the weekends (Table 2). Infrequent Internet gambling was also associated with Internet access either via an Internet café portal or cellular phone. Moreover, such gambling practices were associated with an increased likelihood for utilizing the Internet for the purposes of game playing, chat-room use, and/or retrieving sexual information. No significant association was found between infrequent Internet gambling and utilizing the Internet for the purposes of accessing mass media sites and/or purchases of goods (Table 3).

**Table 1.** Characteristics of the study sample according to Internet gambling practices

	Overall Internet gambling (*n* = 281)		Frequent Internet gambling (*n* = 109)		Infrequent Internet gambling (*n* = 172)		No Internet gambling (*n* = 524)
	*n* (%)	*p*	*n* (%)	*p*	*n* (%)	*p*	*n* (%)
Male gender	159 (56.6)	0.001†	68 (62.4)	0.0007†	98 (52.9)	0.054†	233 (44.5)
Age		<0.0001‡		<0.0001‡		<0.0001‡	
13–14.9 years	86 (30.6)		34 (31.2)		52 (30.2)		162 (30.9)
15–15.9 years	130 (46.3)		51 (46.8)		79 (45.9)		144 (27.5)
16–17.9 years	65 (23.1)		24 (22.0)		41 (23.8)		218 (41.6)
Rural (vs. urban) residence	63 (22.4)	0.818†	21 (19.3)	0.933†	42 (24.4)	0.806†	110 (21.0)
Parental marital (vs. divorced) status	226 (80.4)	0.763†	87 (79.8)	0.701†	139 (80.8)	0.896†	347 (66.2)
Paternal age (mean years ± SD)	45.8 ± 5.6	0.921§	46.1 ± 5.5	0.608§	45.6 ± 5.6	0.810§	45.8 ± 5.3
Paternal educational status							
Secondary schooling	102 (36.3)	0.911†	40 (36.7)	0.947†	62 (36.0)	0.917†	164 (31.3)
Tertiary schooling	65 (23.1)	0.687†	24 (22.0)	0.924†	41 (23.8)	0.628†	94 (17.9)
Maternal age (mean years ± SD)	41.8 ± 5.1	0.535§	41.9 ± 5.0	0.731§	41.8 ± 5.2	0.551§	42.1 ± 5.2
Maternal educational status							
Secondary schooling	79 (28.1)	0.731†	28 (25.7)	0.445†	51 (29.6)	0.925†	121 (23.1)
Tertiary schooling	54 (19.2)	0.381†	21 (19.3)	0.436†	33 (19.2)	0.534†	93 (17.7)

† Chi-square test *p*-value.

‡ Likelihood ratio *p*-value.

§ *t*-test *p*-value.

**Table 2.** Characteristics of Internet use according to Internet gambling practices

	Overall Internet gambling (*n* = 281)		Frequent Internet gambling (*n* = 109)		Infrequent Internet gambling (*n* = 172)		No Internet gambling (*n* = 524)
	*n* (%)	*p*	*n* (%)	*p*	*n* (%)	*p*	*n* (%)
Length of Internet use							
0–12 months	60 (21.4)		27 (24.8)		33 (19.2)		208 (39.7)
12–36 months	140 (49.8)	0.480†	54 (49.5)	0.576†	86 (50.0)	0.587†	241 (46.0)
>36 months	81 (28.8)	0.006†	28 (25.7)	0.082†	53 (30.8)	0.012†	75 (14.3)
Average Internet use on weekdays		0.0001‡	<0.0001‡		0.010‡	
0–0.9 hours	34 (12.1)		15 (13.8)		19 (11.0)		116 (22.1)
1–8.9 hours	202 (71.9)		66 (60.6)		136 (79.1)		359 (68.5)
9–20.9 hours	32 (11.4)		18 (16.5)		14 (8.1)		42 (8.0)
≥ 21 hours	13 (4.6)		10 (9.2)		3 (1.7)		7 (1.3)
Average Internet use on weekends		<0.0001‡		<0.0001‡		0.004‡	
0–0.9 hours	33 (11.7)		14 (12.8)		19 (11.0)		93 (17.8)
1–8.9 hours	165 (58.7)		57 (52.3)		108 (62.8)		353 (67.4)
9–20.9 hours	69 (24.6)		30 (27.5)		39 (22.7)	70 (13.4)
≥ 21 hours	14 (5.0)		8 (7.3)		6 (3.5)		8 (1.5)
Internet addiction test							
Normal use	196 (69.7)		65 (59.6)		131 (78.4)		445 (84.9)
Borderline addictive use	73 (26.0)	<0.0001†	37 (33.9)	<0.0001†	36 (21.6)	0.029†	75 (14.3)
Internet addictive behavior	12 (4.3)	0.0002†	7 (6.4)	<0.0001†	5 (3.7)	0.021†	4 (0.8)

† Chi-square test *p*-value.

‡ Likelihood ratio *p*-value.

**Table 3.** Locations of Internet access and scope of Internet sites used among adolescents according to Internet gambling practices

	Overall Internet gambling (*n* = 281)		Frequent Internet gambling (*n* = 109)		Infrequent Internet gambling (*n* = 172)		No Internet gambling (*n* = 524)
	*n* (%)	*AOR (95% CI)*	*n* (%)	*AOR (95% CI)*	*n* (%)	*AOR (95% CI)*	*n* (%)
Locations of access							
Own home	274 (97.5)	2.00 (0.85–4.69)	108 (99.1)	5.53 (0.74–4.14)	166 (96.5)	1.42 (0.57–3.53)	498 (95.0)
Friend’s home	241 (85.8)	1.94 (1.30–2.87)	92 (84.4)	1.76 (1.00–3.08)	149 (86.6)	2.13 (1.31–3.46)	397 (75.8)
Internet café	146 (52.0)	2.13 (1.56–2.91)	67 (61.5)	2.97 (1.90–4.64)	79 (45.9)	1.71 (1.18–2.48)	170 (32.4)
Cellular phone	135 (48.0)	1.62 (1.19–2.19)	56 (51.4)	1.77 (1.16–2.72)	79 (45.9)	1.51 (1.08–2.16)	186 (35.5)
Sites accessed E-mail	259 (92.2)	1.55 (0.92–2.61)	100 (91.7)	1.52 (0.72–3.20)	159 (92.4)	1.55 (0.82–2.90)	465 (88.7)
Mass media	180 (64.1)	1.13 (0.84–1.54)	67 (61.5)	0.93 (0.60–1.42)	115 (66.9)	1.29 (0.89–1.86)	322 (61.4)
Chat-rooms	268 (95.4)	2.57 (1.31–4.82)	104 (95.4)	2.72 (1.06–7.00)	164 (95.4)	2.53 (1.18–5.44)	468 (89.3)
Game-playing	257 (91.5)	6.84 (4.23–11.07)	101 (92.7)	7.79 (3.60–16.84)	156 (90.7)	6.29 (3.55–11.16)	322 (61.4)
Sexual information	134 (47.7)	1.99 (1.42–2.81)	60 (55.0)	2.57 (1.59–4.15)	74 (43.0)	1.69 (1.14–2.53)	162 (30.9)
Purchases	151 (53.7)	1.38 (0.99–1.91)	57 (52.3)	1.72 (1.03–2.87)	94 (54.6)	1.24 (0.85–1.80)	270 (51.5)

AOR: Adjusted odds ratio for age and gender; 95% CI: 95% Confidence Interval.

The occurrence of both borderline addictive Internet use and Internet addictive behavior was significantly greater among adolescents with infrequent Internet gambling as compared to their normal counterparts. Specifically, approximately one fifth (21.6%; *n* = 36) of adolescents with infrequent Internet gambling reported concomitant borderline addictive Internet use, while 3.7% (*n* = 5) reported Internet addictive behavior (Table 2). While infrequent Internet gambling was associated with an increased risk for presenting with an abnormal Total Strengths & Difficulties score, these were limited to the presentation of abnormal conduct problems (Table 4).

### Frequent Internet gambling practices among adolescents

The prevalence of frequent Internet gambling among the study sample was 13.5% (*n* = 109). Frequent Internet gamblers were more likely to engage in extended hours of Internet use both during the weekdays and weekends (Table 2). Moreover, frequent Internet gambling was associated with a threefold increase in the likelihood of accessing the Internet via an Internet café portal. Members of this group were also significantly more likely to utilize the Internet via their cellular phone (Table 3). Adolescent frequent Internet gambling was associated with Internet use for the purposes of game-playing, chat-room use, and retrieval of sexual information. Moreover, such practices were also significantly associated with utilization of the Internet for purchasing goods (Table 3).

One third of adolescents who participated in frequent Internet gambling reported borderline addictive Internet use. Moreover, members of this group were significantly more likely to present with Internet addictive behavior as compared to their normal counterparts (Table 2). Frequent Internet gambling was associated with a notable diminishment of Total Strengths and Difficulties score. In particular, adolescent frequent Internet gambling was associated with abnormal conduct problems and emotional symptoms. Furthermore, such gambling practices were significantly associated with abnormal hyperactivity scores (Table 4).

**Table 4.** Component and total Strengths & Difficulties (SDQ) scores among adolescents according to Internet gambling practices

	Overall Internet gambling (*n* = 281)		Frequent Internet gambling (*n* = 109)		Infrequent Internet gambling (*n* = 172)		No Internet gambling (*n* = 524)
	*n* (%)	*AOR (95% CI)*	*n* (%)	*AOR (95% CI)*	*n* (%)	*AOR (95% CI)*	*n* (%)
Emotional symptoms							
Borderline	12 (4.3)	1.37 (0.65–2.89)	5 (4.6)	1.72 (0.61–4.86)	7 (4.1)	1.21 (0.49–2.96)	20 (3.8)
Abnormal	25 (8.9)	1.78 (1.02–3.11)	12 (11.0)	2.47 (1.19–5.12)	13 (7.6)	1.42 (0.72–2.82)	34 (6.5)
Conduct problems							
Borderline	43 (15.3)	1.43 (0.93–2.20)	17 (15.6)	1.61 (0.88–2.94)	26 (15.1)	1.35 (0.82–2.25)	65 (12.4)
Abnormal	47 (16.7)	3.26 (2.00–5.32)	26 (23.8)	5.20 (2.87–9.42)	21 (12.2)	2.30 (1.27–4.16)	31 (5.9)
Hyperactivity							
Borderline	28 (10.0)	2.62 (1.44–4.75)	15 (13.8)	4.12 (2.00–8.52)	13 (7.6)	1.94 (0.94–4.00)	21 (4.0)
Abnormal	12 (4.3)	1.49 (0.70–3.17)	8 (7.3)	2.98 (1.23–7.22)	4 (2.3)	0.73 (0.24–2.21)	18 (3.4)
Peer problems							
Borderline	46 (16.4)	1.40 (0.92–2.12)	18 (16.5)	1.39 (0.78–2.49)	28 (16.3)	1.42 (0.87–2.31)	68 (13.0)
Abnormal	15 (5.3)	2.44 (1.08–5.48)	6 (5.5)	2.35 (0.83–6.69)	9 (5.2)	2.50 (1.00–6.29)	11 (2.1)
Prosocial							
Borderline	34 (12.1)	1.19 (0.74–1.92)	11 (10.1)	1.01 (0.50–2.05)	23 (13.4)	1.33 (0.78–2.28)	50 (9.5)
Abnormal	26 (9.2)	1.08 (0.64–1.85)	15 (13.8)	1.56 (0.81–3.01)	11 (6.4)	0.78 (0.38–1.58)	41 (7.8)
Total SDQ							
Borderline	38 (13.5)	2.02 (1.26–3.24)	14 (12.8)	2.14 (1.10–4.18)	24 (14.0)	2.02 (1.17–3.48)	43 (8.2)
Abnormal	23 (8.2)	4.00 (1.97–8.13)	14 (12.8)	6.90 (3.04–15.66)	9 (5.2)	2.52 (1.04–6.07)	13 (2.5)

AOR: Adjusted odds ratio for age and gender; 95% CI: 95% Confidence Interval.

The multivariate regression analyses conducted indicated that the single independent predictor associated with Internet addictive behavior was Internet gambling (intercept: 1.73; standard error: 0.70; AOR: 5.66; 95% CI: 1.45– 22.15). It is notable that neither the locations of Internet access nor the scope of sites utilized were independently associated with Internet addictive behavior following adjustment for the effect of gambling practices.

## DISCUSSION

The present study assessed the prevalence of Internet gambling practices, as well as its impact upon psychosocial development, among adolescents. In addition, the study evaluated the association between the frequency of gambling practices and development of Internet addictive behavior among youth. The findings indicated that approximately one third of the youth population examined participated in Internet gambling practices. Moreover, approximately one fifth of adolescents engaged in such practices infrequently, while 13.5% participated in Internet gambling frequently. The observed prevalence of Internet gambling is markedly greater than that reported among other adolescent populations (Hardoon, Gupta & Derevensky, 2004; Molde et al., 2009; Moodie & Finnigan, 2006; Petry & Weinstock, 2007). It is of note that the observed elevated rates of Internet gambling among the study population may be attributed to socio-cultural factors of the region. In particular, gambling practices among adults are legally permissible in the examined region and may thus diminish social barriers among adolescents for participating in such practices both in real-life and cyber venues.

Similarly to previous reports (Dickson et al., 2002; Griffiths et al., 2009; Molde et al., 2009; Tsitsika, Critselis, Janikian, Kormas & Kafetzis, 2011), the study findings indicated that both infrequent and frequent Internet gambling were more often adopted by male adolescents. Moreover, the findings indicated that adolescents participating in gambling patterns were more likely to utilize the Internet extensively both during the weekdays and weekends. The observed preference of male adolescents to engage in Internet gambling may be confounded by the fact that female adolescents are less likely to utilize the Internet frequently (Tsitsika et al., 2009a), particularly for purposes other than social communication (Tsitsika et al., 2009b), and may thus access less often gambling sites (Tsitsika et al., 2011).

The study findings indicated that engagement in Internet gambling, regardless of the frequency of use, was significantly associated with marked psychosocial and emotional maladjustment. These findings contradict previous reports indicating that Internet gambling behavior does not impact the psychosocial development of adolescents (Tsitsika et al., 2011). However, it is of note that frequent Internet gambling, in particular, was associated with the development of adverse emotional symptoms among the adolescent population examined. Emotional symptoms, including depression and suicidal ideation, have been previously correlated with gambling practices among youth (Bakken, Gotestam, Grawe, Wenzel & Oren, 2009; Messerlian, Derevensky & Gupta, 2005). It is inferred from the study findings that augmentation in the frequency of gambling practices is positively associated with the development of emotional symptoms which may consequently adversely affect adolescents’ functionality and performance both within their academic and social environments.

Moreover, engagement in Internet gambling practices, regardless of frequency of use, was significantly associated with marked conduct problems and curtailed peer relations. The frequency of gambling behavior among adolescents was positively correlated with the risk of developing conduct problems. Similar findings have been reported previously indicating that adolescents who engage in Internet gambling are more likely to display aggressive behavior (Hardoon et al., 2004; Ko, Yen, Liu, Huang & Yen, 2009). Furthermore, Internet gambling among adolescents was associated with notable peer problems, particularly among those engaging in frequent Internet gambling. While an etio-logical association has not been established, adolescent gambling has been previously correlated with introversion, social isolation, and consequent curtailed social networks (Messerlian et al., 2005).

Finally, the study findings indicated that the likelihood of Internet addictive behavior among adolescents who participated in Internet gambling was increased in excess of fivefold. Furthermore, among all parameters assessed, including locations of Internet access and scope of Internet sites visited, the study findings indicated that the most significant independent predictor of Internet addictive behavior among adolescents was Internet gambling. While Internet gambling may predispose the development and manifestation of pathological gambling behaviors (Griffiths, 1999, 2003; Griffiths & Parke, 2002; Griffiths & Wood, 2000; LaRose, Mastro & Eastin, 2001), an etiological association has not been yet defined (Griffiths, 2003; Wölfling et al., 2009).

It is upheld that the Internet may provide a readily accessible medium for adopting gambling patterns, and potentially developing and exhibiting impulsive and/or addictive behavioral patterns such as pathological gambling (Recupero, 2008; Sun et al., 2009), among adolescents. Moreover, it is plausible that the sustainment of such behaviors through this medium may in turn lead to the development of Internet addictive behavior. Thus, adolescents who participate even infrequently in Internet gambling have an increased likelihood for presenting with Internet addictive behavior. However, additional longitudinal studies are necessary in order to clarify whether adolescents who participate in Internet gambling and concomitantly present with Internet addictive behavior exhibit multiple independent addictive behaviors (Pallanti et al., 2006) or whether Internet addictive behavior constitutes the result of problematic Internet gambling behaviors.

The limitations of the present study include that due to the cross-sectional study design applied, and consequent lack of knowledge of the temporal sequence and causality of events, the potential etiological association between Internet gambling and Internet addictive behavior cannot be ascertained. Moreover, the study design did not permit for the assessment of pre-existing psychological factors which may confound the association between Internet gambling and Internet addictive behavior, and/or the persistence of such behaviors over time. In addition, the extrapolation of the study findings are limited to adolescents participating in gambling practices over the internet, as opposed to real-life venues. Due to the self-reported assessment tools utilized, both a selection and a reporting bias may have been introduced regarding both the frequency of Internet gambling and/or Internet addictive behavior. However, it is upheld that such a bias would lead to an underestimation of the true prevalence of both Internet gambling practices and Internet addictive behavior. Finally, due to the lack of a gold standard for measuring addictive Internet behaviors, it is uncertain whether the observed high IAT rates may be a result of elevated false-positive reporting rates. The strengths of the present study include that it assessed the prevalence, as well as psychosocial and emotional characteristics, associated with Internet gambling among a substantial sized cohort of adolescents. In addition, the present study constitutes one of few which evaluated the association between Internet gambling practices, according to both overall and frequency of use, and Internet addictive behavior.

## CONCLUSIONS

In conclusion, approximately one third of Cypriot adolescents participate in Internet gambling. Such gambling practices are associated with notable psychosocial and emotional maladjustment, including emotional symptoms, conduct problems, and deterred peer relations. Adolescents who participate in Internet gambling, regardless of the frequency of use, have an augmented likelihood for presenting with Internet addictive behavior. Finally, among all parameters of Internet use assessed, including locations of Internet access and scope of Internet sites visited, the study findings indicated that the sole independent predictor of Internet addictive behavior among adolescents is Internet gambling. Additional longitudinal studies are needed in order to elucidate the underlying developmental and potential causal relationships between Internet gambling and Internet addictive behavior among adolescents.
